# Gut Microbiome Disruption Following SARS-CoV-2: A Review

**DOI:** 10.3390/microorganisms12010131

**Published:** 2024-01-09

**Authors:** Elda Righi, Ilaria Dalla Vecchia, Nina Auerbach, Matteo Morra, Anna Górska, Concetta Sciammarella, Lorenza Lambertenghi, Elisa Gentilotti, Massimo Mirandola, Evelina Tacconelli, Assunta Sartor

**Affiliations:** 1 IMID Laboratory, Department of Diagnostics and Public Health, Infectious Diseases Division, University of Verona, 37134 Verona, Italyanna.gorska@univr.it (A.G.); concetta.sciammarella@univr.it (C.S.); evelina.tacconelli@univr.it (E.T.); 2 Microbiology Unit, Udine University Hospital, 33100 Udine, Italy; assunta.sartor@asufc.sanita.fvg.it

**Keywords:** microbiome, COVID-19, beneficial symbionts, opportunistic pathogens, post-COVID-19 syndrome

## Abstract

COVID-19 has been associated with having a negative impact on patients’ gut microbiome during both active disease and in the post-acute phase. In acute COVID-19, rapid alteration of the gut microbiome composition was observed, showing on one side a reduction in beneficial symbionts (e.g., *Roseburia*, *Lachnospiraceae*) and on the other side an increase in opportunistic pathogens such as *Enterococcus* and *Proteobacteria*. Alpha diversity tends to decrease, especially initially with symptom onset and hospital admission. Although clinical recovery appears to align with improved gut homeostasis, this process could take several weeks, even in mild infections. Moreover, patients with COVID-19 post-acute syndrome showed changes in gut microbiome composition, with specific signatures associated with decreased respiratory function up to 12 months following acute disease. Potential treatments, especially probiotic-based therapy, are under investigation. Open questions remain on the possibility to use gut microbiome data to predict disease progression and on potential confounders that may impair result interpretation (e.g., concomitant therapies in the acute phase; reinfection, vaccines, and occurrence of novel conditions or diseases in the post-acute syndrome). Understanding the relationships between gut microbiome dynamics and disease progression may contribute to better understanding post-COVID syndrome pathogenesis or inform personalized treatment that can affect specific targets or microbiome markers.

## 1. Introduction

The COVID-19 pandemic shed light on the intricate relationship between infectious diseases and the human gut microbiome. Like influenza, SARS-CoV-2 has also been associated with having a negative impact on patients’ gut microbiome, especially during acute disease [1]. Current research suggests that COVID-19 can significantly impact the gut microbiome composition [2]. Understanding these connections is key to unraveling the mechanisms of the disease and may contribute to the development of novel therapeutic strategies and diagnostics [1,2,3].

Changes in the human gut microbiome composition exist both in acute and during lingering phases of the disease. In the acute phase, studies have indicated that COVID-19 patients often exhibit alterations in their gut microbial composition, characterized by a decrease in the abundance of beneficial microbes and an increase in potentially harmful species [4]. These disruptions may contribute to the systemic inflammation and immune dysregulation seen in severe cases of COVID-19, potentially influencing disease severity. Moreover, as individuals transition to the post-acute phase (defined by the WHO as the development of new symptoms 3 months after the initial SARS-CoV-2 infection, with these symptoms lasting for at least 2 months with no other explanation) [5], persistent symptoms such as gastrointestinal distress, fatigue, and neurological issues have been reported [6,7,8,9]. The gut microbiome’s role in post-COVID syndrome (PCS) is still under investigation. Nevertheless, understanding microbiome changes during acute and PCS is paramount not only for improving our comprehension of the disease but also for devising targeted interventions that could aid in disease recovery and mitigate long-term health implications in specific populations.

In this review, we present current knowledge on COVID-19 effects on the gut microbiome, both in acute and prolonged disease. We also included original data from our group regarding a longitudinal study of microbiome changes in a group of healthcare workers following SARS-CoV-2 infection.

## 2. Methods

### 2.1. Systematic Review

A Medline review was performed using the terms “acute COVID-19”, “long-COVID”, “post-acute COVID-19 syndrome”, “post COVID syndrome”, and “gut microbiome” from 1 February 2020 to 30 March 2023. Relevant original articles on the gut microbiome and COVID-19 were included. We considered the literature on acute COVID-19 and dysbiosis, as well as studies reporting data on patients recovering from COVID-19 and collecting stool samples over time (e.g., longitudinal studies) and from patients suffering from PCS. Studies including at least 5 subjects were considered with no restriction on the technology used (e.g., 16S rRNA gene sequencing or whole genome sequencing, WGS) or the type of analysis performed (e.g., microbiome composition and functional analysis).

Systematic review and meta-analysis were not performed due to the high heterogeneity of the studies in terms of duration of follow-up, type of patients, and disease severity.

### 2.2. Longitudinal Study Methodology

Our group performed a prospective longitudinal study involving 6 healthcare workers with COVID-19. Clinical characteristics, inflammation profiles, and bacterial relative abundance before and after SARS-CoV-2 infection were analyzed (unpublished data). The study protocol was approved by the institutional review board at Verona University Hospital (CO-BIOME study, IRB number 2906). Informed consent was provided by all study participants. The methods for stool processing and 16S rRNA gene sequencing have been previously reported [4]. SARS-CoV-2 infection was diagnosed by real time multiplex reverse transcription polymerase chain reaction (multiplex RT-PCR) for simultaneous detection of three different SARS-CoV-2 targets (E, N and RdRP genes) from nasopharyngeal and oropharyngeal swabs using Allplex 2019-nCoV assay kit (Seegene, Seoul, Republic of Korea). Stool samples were stored at −80 °C until DNA extraction. DNA was isolated with the Norgen Stool DNA Isolation Kit (Norgen Biotek, Thorold, ON, Canada) with the addition of mechanical lysis and following the manufacturer’s instructions. DNA samples were subsequently quantified by a spectrophotometer (Maestrogen, Brumath Cedex, France) and normalized to a concentration of 50 ng/μL. The 16S rRNA gene V1-V3 region was amplified using previously defined primers and underwent paired-end sequencing using version 3 (300 × 2) chemistry on the MiSeq instrument [Illumina] according to manufacturer’s instructions [10]. To measure stool positivity for SARS-CoV-2, the Xpert^®^ Xpress SARS-CoV-2 test (Cepheid, Maurens-Scopont, France) was used. This automated test performs real-time RT-PCR analysis amplifying sequences of the envelope (E) and nucleocapsid (N2) genes and provides positive results when both targets, or the N2 target only, are detected (cycle threshold < 45 and endpoint above the minimum setting). The analyses are reported at a genus level showing bacteria with abundance ≥ 0.1%. Alpha (Shannon’s diversity index) and beta diversity metrics (Jaccard distance, weighted UniFrac distance) were used to describe the gut microbiome composition.

To better define inflammatory profiles that could be representative of the general condition of the gut microbiome, a pro-inflammatory (PI) to anti-inflammatory (AI) ratio (PI/AI ratio) was used. The genera were classified as PI or AI using over 700 abstracts from an automatized search of the PubMed database as described in Supplementary Methods. The AI/PI ratio was classified into 5 profiles, namely highly AI, moderately AI, low PI, moderately PI, and highly PI, to reflect the overall scale of the dysbiosis in the gut microbiome.

#### Bioinformatics and Statistical Analysis

The quality of reads was controlled using FastQC. The pre-processing, trimming, filtering, and merging was carried out with Cutadapt [11]. Reads were subsequently aligned against the SILVA ARB 16S rRNA v. SSU 138 database [12] with minimap2 [13]. Next, the taxonomic profiles were computed using MEGAN6 [14] and exported into csv files on multiple taxonomic levels in a summarized manner. The downstream analysis and visualizations were carried out in Python3.8 with matplotlib v. 3.2.1 [15] and scipy [16]. Computation of the weighted UniFrac [17] was carried out in Python3.8 using a scikit-bio [18] and ete3 [19] packages. Median and ranges were for continuous variables, and count and percentages for nominal variables. Kruskal–Wallis test was used for comparing independent groups. A two-tailed α error of 0.05 was used in all analyses.

## 3. Changes in Gut Microbiome during and Following Acute COVID-19

Several microbiome changes have been reported in association with COVID-19, including a reduction in microbial diversity, a shift in the relative abundance of certain bacterial species (e.g., favoring opportunistic pathogens vs. symbionts), and an overall imbalance in the gut ecosystem (Table 1).

The majority of the studies investigated the changes in gut microbiome composition in hospitalized patients with acute COVID-19, while very few studies were performed among asymptomatic patients or before and after recovery.

### 3.1. Acute COVID-19

Cheng et al. (2022) performed a meta-analysis of 16S rRNA microbial data encompassing 14 studies to report microbiome changes associated with COVID-19 over time [27]. The focus of the study was to investigate and analyze the gut microbiota in individuals with COVID-19 and in those who had recovered compared with non-COVID-19 individuals. The total study population consisted of 1385 individuals, with a broad geographic representation including Asia, America, and Europe. Additionally, the study explored potential differences in gut microbiota associated with the severity of COVID-19, specifically comparing non-severe (both inpatient and outpatient) and severe COVID-19 patients. Among these studies, 11 did not adjust for covariates such as age, gender, and antibiotic treatment when assessing the differential abundance of gut bacterial taxa or pathways between groups, therefore posing an important limitation in the interpretation of the results. The follow-up time significantly varied between studies, showing a maximum duration of up to 6 months. The main findings showed that COVID-19 patients undergo substantial modifications in gut microbiota composition during acute vs. recovery phases and have distinct patterns compared with SARS-CoV-2 non-infected individuals. Specifically, COVID-19 patients consistently exhibited diminished microbial diversity, accompanied by an increased prevalence of opportunistic pathogens such as *Streptococcus*, *Enterococcus*, and *Corynebacterium*. When comparing COVID-19 patients across levels of severity, the results showed a non-significant decrease in alpha diversity in severe compared with non-severe COVID-19 patients. Throughout the recovery phase, COVID-19 patients consistently maintained a lower level of gut bacterial diversity as indicated by Shannon’s diversity index, Pielou’s evenness, and Faith’s phylogenetic diversity compared to controls. Furthermore, the diversity had a tendency to rebound in recovered patients who were relatives of COVID-19 patients during the acute phase of the disease, indicating a transition to a more favorable ecological state following the clearance of SARS-CoV-2 [27]. The diversity within the study populations, however, was remarkable and attributable to several factors, including a substantial variability in gut microbiota across various demographics, lifestyles, dietary habits, as well as the disparities in study protocols, data analysis, and statistical techniques. It is also worth mentioning that most of the studies had a cross-sectional design that did not clearly enable them to evaluate the changes over time during the acute phase of the disease.

Other studies highlighted distinct alterations in human gut microbiome composition associated with acute COVID-19 (Table 1). Overall, the analysis of microbial differential abundance highlighted a depletion in anti-inflammatory butyrate-producing bacteria (BPB) and an enrichment of taxa with pro-inflammatory properties during acute COVID-19.

### 3.2. COVID-19 Severity and Gut Microbiome Changes

Gaibani et al. (2021) analyzed fecal samples from 57 critically ill COVID-19 patients, showing that patients had a significant reduction in alpha diversity compared to 100 healthy controls [20]. The composition of the gut microbiota in COVID-19 patients showed deep structural changes compared with non-COVID-19 critically ill patients, including alterations in the dominant phyla (Firmicutes and Bacteroidetes) and subdominant components such as Actinobacteria and Synergistetes. Dysbiosis was characterized by reduced diversity, depletion of beneficial commensals (e.g., short-chain fatty acid—SCFA—producers from *Lachnospiraceae* and *Ruminococcaceae* families), and enrichment in opportunistic pathogens or pathobionts. The over-representation of *Enterococcus* was found to be closely related to ICU admission and the development of bloodstream infections [20]. Our group has also previously highlighted the role of *Enterococcus* in association with antibiotic use and severity in COVID-19 patients [4]. Another small study conducted in Italy by Mazzarelli et al. (2021) sought to examine the potential impact of SARS-CoV-2 pneumonia on the gut microbiota of fifteen hospitalized patients in comparison with eight controls who were admitted for pneumonia during the same time period [21]. The study showed that individuals with SARS-CoV-2 pneumonia, especially ICU patients, exhibited notable alterations compared with non-ICU patients and with controls, showing a reduced microbial richness quantified by the Chao1 index. Furthermore, Proteobacteria were elevated, while Spirochaetes and Fusobacteria were reduced in non-ICU COVID-19 patients. At the family level, several potential pathogenic bacterial families were significantly increased in non-ICU COVID-19 patients compared with controls, including *Peptostreptococcaceae*, *Enterobacteriaceae*, *Staphylococcaceae,* and *Vibrionaceae*, while *Nitrospiraceae*, *Propionibacteriaceae*, *Aeromonadaceae*, *Moraxellaceae*, and *Mycoplasmataceae* were reduced. Differences in the microbiota composition for intensive care- and ward-based COVID-19 patients were not statistically significant at the phylum level.

Hierarchical clustering analysis revealed distinct microbial signatures for critically ill compared to ward-based COVID-19 patients and controls. These findings suggest that ICU admission for COVID-19 may lead to specific alterations in the gut microbiota that differ from those observed in patients not admitted to the ICU [21]. Another cross-sectional study by Moreira-Rosário et al. (2021) aimed to investigate the relationship between gut microbiota composition and COVID-19 severity, analyzing a total of 111 fecal samples [22]. The authors compared the gut bacterial composition and clinical variables (such as age, sex, BMI, smoking status, comorbidities, symptoms, laboratory parameters, and treatment) according to COVID-19 severity (e.g., asymptomatic/mild, moderate, and severe according to the WHO Clinical Progression Scale). Compared with moderate or severe cases, patients who were asymptomatic or with mild disease exhibited higher alpha diversity (Shannon Index). Specific bacterial families decreased with disease severity (e.g., *Lachnospiraceae*, *p* < 0.001, False Discovery Rate, FDR-corrected), whereas *Proteobacteria* increased with disease severity (*p* < 0.001; FDR-corrected). The setting of recovery (e.g., outpatient setting, ward, or ICU) corresponded to distinct gut microbiome signatures, with *Proteobacteria* abundance rising with severity. Multivariate analysis indicated that increased C-reactive protein (CRP) levels and lower microbiome diversity were significantly associated with severe COVID-19 (aOR = 2.8S, 95% CI 1.09–7.41), while risk of severe disease progression was shown in terms of Shannon diversity index < 2.25 (*p* = 0.032). Additionally, SARS-CoV-2 RNA was detected in 40% of fecal samples, more frequently in males, but no association emerged between fecal SARS-CoV-2 positivity and disease severity or microbiome composition [22]. Another small cross-sectional study by Khan et al. (2021) assessed the correlation between gut microbiota and the immune response in COVID-19 patients [23]. The study included four groups: controls (healthy subjects, *n* = 10), asymptomatic SARS-CoV-2 patients (*n* = 10), mild COVID-19 (*n* = 10), and severe COVID-19 (*n* = 10). A decrease in gut bacterial diversity was noted according to disease severity, while a shift in bacterial composition was more pronounced in severe COVID-19 patients, although a decreased Firmicutes/Bacteroidetes ratio was noted in both mild and severe disease. Linear Discriminant Analysis showed that several bacterial groups, including *Faecalibacterium prausnitzii*, *Roseburia*, *Prevotella*, and *Bacteroides plebeius,* were reduced in COVID-19 patients compared with controls, while other genera, such as *Bifidobacterium*, were increased, especially in severe cases. COVID-19 patients with severe disease had elevated levels of the inflammatory cytokine IL-21 compared with mild disease and healthy individuals (*p* = 0.01). Nevertheless, there were no significant differences in TNF-α and INF-γ levels among the groups [23].

De Nies et al. (2023) used high-resolution systematic multi-omic analyses to investigate the gut microbiome in asymptomatic-to-moderate COVID-19 patients [28]. Compared to controls, COVID-19 patients, even with mild disease, showed an increase in virulence factors and antimicrobial resistance genes that are encoded and expressed by Acidaminococcaceae and Erysipelatoclostridiaceae.

A study by Reinold et al. (2021) revealed lower bacterial richness in 117 SARS-CoV-2 positive patients with diverse grades of disease severity when compared to 95 controls [24]. Only 3% of SARS-CoV-2 positive and negative patients had received antibiotics prior to sampling. Positive patients showed reduced bacterial richness, while the Shannon diversity index and Pielou’s evenness displayed no significant differences compared with controls. Principal Coordinates Analysis (PCA) demonstrated dissimilarities between the two groups, indicating altered gut microbiota composition in SARS-CoV-2 patients. Additionally, the relative abundance of Proteobacteria and Bacteroidetes phyla was significantly higher in SARS-CoV-2 patients compared with controls, whereas Actinobacteria was less abundant [24]. Among the 20 most abundant genera, 5 showed significant differences between the groups, with *Bacteroides* being more prevalent in SARS-CoV-2 positive patients, while *Bifidobacterium*, *Collinsella*, *Streptococcus*, and *Corynebacterium* were more abundant in SARS-CoV-2 negative patients. Further analysis using LDA of effect size confirmed these findings, highlighting that several bacterial taxa could act as distinguishing markers between the two groups, with *Enterobacteriaceae* and *Campylobacteraceae* being characteristic of positive patients [16]. Additionally, differences in the gut microbiome were explored in relation to COVID-19 severity. Patients with severe and critical COVID-19 had lower levels of BPB. *Faecalibacterium* and *Roseburia* were identified as key genera that discriminated between severe/critical and non-severe COVID-19 patients and were associated with an interconnected cluster of bacteria characterized by anti-inflammatory properties and SCFA production such as *Dorea*, *Blautia*, *Coprococcus*, *Lachnospira*, and others [24].

### 3.3. Gut Microbiome in Patients Recovering from COVID-19

A case–control study by Newsome et al. (2021) analyzed the microbial community structure of fecal samples of 93 patients, including 50 SARS-CoV-2 infected patients, 9 SARS-CoV-2 recovered patients, and 34 SARS-CoV-2 non-infected controls [25]. Most patients were of African American origin. The actively infected group had a higher prevalence of comorbidities, particularly diabetes and hypertension, and a significant portion received antibiotic treatment. The gut microbiome of SARS-CoV-2 patients was characterized by an increased relative abundance of *Campylobacter* and *Klebsiella*. Clustering showed clear differences between infected patients, controls, and recovered patients independently of antibiotic exposure. Despite different treatments, all groups showed a similar Shannon diversity (*p* = 0.78, FDR corrected). Recovered patients’ microbiome profiles were closer to the non-infected controls. The presence of detectable virus in feces was associated with differences in the microbial community, with higher relative abundances shown by *Comamonas*, *Sphaerochaeta*, *Synergistes*, *Klebsiella*, and *Agathobacter* among qPCR-positive samples. Conversely, in qPCR-negative samples, *Pseudoclavibacter*, *Cutibacterium*, *Mycoplasma*, and *Phocea* were enriched.

Longitudinal studies assessing clinical and gut microbiome recovery in outpatients are lacking. Another small study by Kim et al. (2021) aimed to investigate the changes in gut microbiota in asymptomatic or mild COVID-19 outpatients [26]. The study design involved the analysis of fecal samples from 12 patients with COVID-19, who were assessed twice during the study period. The first fecal sample was collected during SARS-CoV-2 infection and the second after recovery. The median duration from diagnosis to fecal sample examination was 38 days, with an average follow-up period of 53 days. Alpha diversity, richness, and evenness showed no significant differences between infected and recovery state. Pielou’s evenness was notably higher in the recovered state. Shannon’s diversity increased in the infected state for most patients, though not reaching statistical significance. Beta diversity analysis revealed significant compositional differences between the two periods. Bacteroidetes were depleted in the infected state, whereas the order Actinomycetales was reduced during recovery. The Firmicutes/Bacteroidetes ratio was substantially higher in the infected state but decreased during recovery, indicating a shift towards a less impaired environment. Comparisons with healthy controls showed that the gut microbiota of recovered patients was more similar to a healthy gut microbiome compared with the infected state. Bacteroidetes were more abundant during recovery. Some genera within *Proteobacteria* and *Actinobacteria* were positively associated with COVID-19, while genera linked to SCFA production were diminished in COVID-19 patients compared with healthy controls [26].

## 4. Longitudinal Study of Microbiome Changes: A Case Series

**Figure 1:**
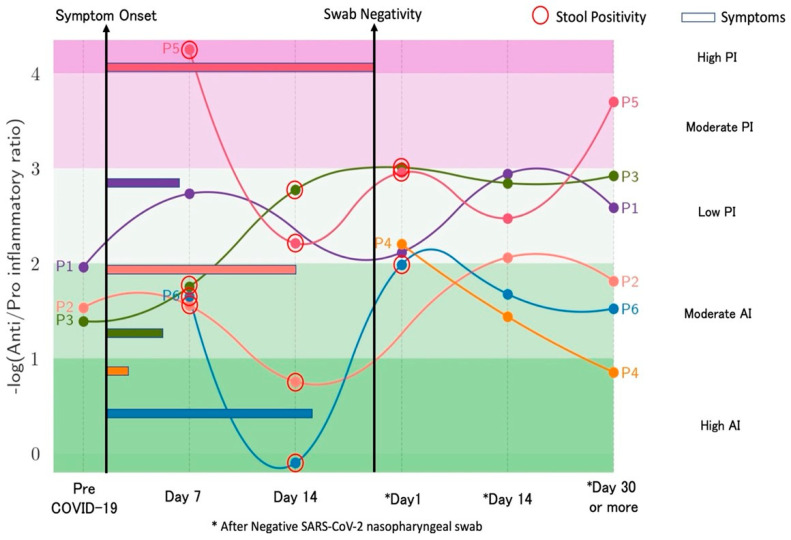
Clinical status and trends of inflammation ratio over time. Trajectories of study participants are shown prior (P1, 2, 3) and before/after (all patients) SARS-CoV-2 are shown. Swab negativity indicates no isolation of SARS-CoV-2 by nasopharyngeal swab. Red circles identify stool positivity and colored bars indicate the duration of COVID-19 related symptoms. The spectrum of colors (values between −0.2 and 4.4 according to the anti/proinflammatory ratio) identifies 5 levels of inflammation, namely highly anti-inflammatory (AI), moderately AI, low proinflammatory (PI), moderately PI, highly PI.

**Figure 2:**
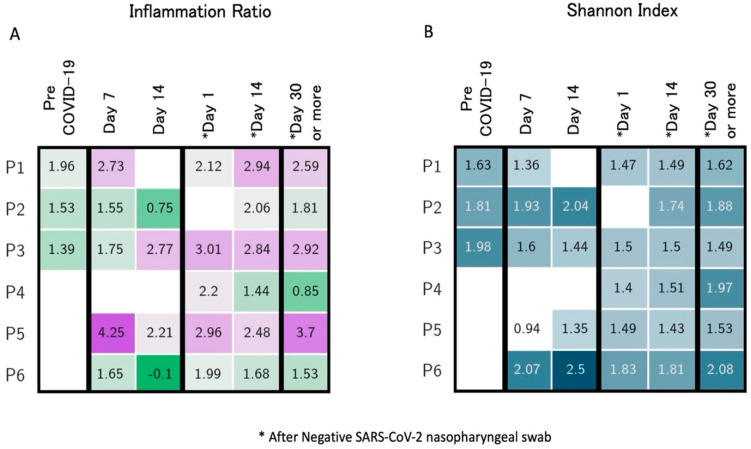
Inflammation ratio (**A**) and Shannon Diversity Index (**B**) longitudinal trends. (**A**). Lower coefficients corresponded to reduced inflammatory profiles. High inflammation levels (>3) are reported using dark purple color according to the color scale indicated in Figure 1. (**B**). Low diversity index is considered for value ≤1.5, with darker colors corresponding to increased values. The asterisks indicate stool collection after negativization of SARS-CoV-2 nasopharyngeal swab.

**Figure 3:**
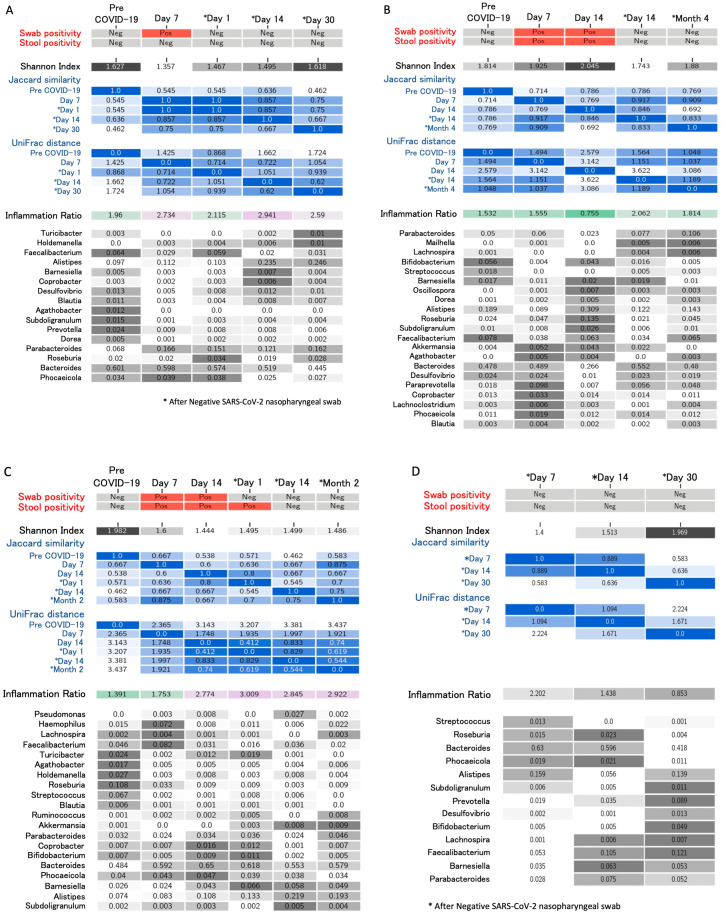

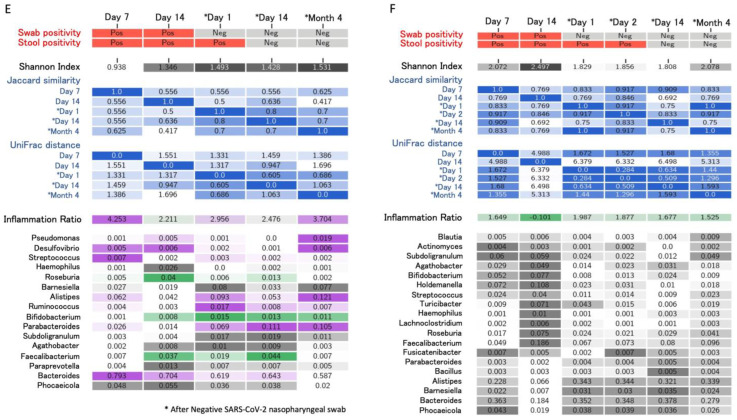
Virological characteristics, alpha and beta indexes, and bacteria relative abundance over time. (**A**–**F**) correspond to patients P1 to P6, respectively. For each patient, swab positivity, stool positivity, Shannon index, Jaccard similarity, UniFrac distance, inflammation ratio, and pathogen relative abundance are reported over time. The asterisks on the days indicate stool collection after negativization of SARS-CoV-2 nasopharyngeal swab. Darker colors correspond to increased values.

A case series of 6 healthcare workers (four males and two females, mean age 28 years old) with COVID-19 is presented. Patients’ follow-up ranged from 6 to 20 weeks after disease onset until January 2021 (unpublished data). Three to six samples (with indication to collect at least one sample by day 14 and after day 30 from swab negativization) were collected for each subject, including a sample before the onset of COVID-19 as part of a biobanking collection among health subjects. Subjects were identified as Patients 1 to 6 (P1 to P6). None of the subjects required hospital admission or received antibiotics in the 30 days before COVID-19 diagnosis. P6 received a 10-day course of hydroxychloroquine starting from day 2 after COVID-19 diagnosis, while the other participants did not undergo any treatment. The median duration of symptoms was 10 days (range, 2 to 20 days). The most frequently reported symptoms were fatigue and smell or taste impairment. All participants, except P3 and P4, complained of at least three symptoms at disease onset. When investigated, two participants (P5 and P6) reported complete resolution of all symptoms, particularly fatigue, only 28 and 40 days after COVID-19 symptom onset, respectively. Figure 1 reports the trend of gut composition for each participant according to the inflammatory levels (AI/PI ratio) over time. All pre-COVID-19 samples were similar, and all showed moderate AI levels; none of the subjects returned to their initial levels but showed higher inflammation compared to the baseline. Most changes in inflammation levels occurred around day 14 since symptom onset and then remained following swab or stool negativization. Inflammation levels as well as Shannon diversity index over time are reported in Figure 2, showing a correspondence between increased inflammation levels (≥3) and low diversity index (≤1.5). The lowest diversity was noted immediately after swab negativization and up to 14 days thereafter (mean Shannon index 1.56 ± 0.05), while an increase in diversity was noted from day 30 following swab negativization (mean Shannon index 1.76 ± 0.10). Alpha diversity was completely restored, returning to pre-COVID-19 values for P1 and P2. Pro-inflammatory levels were maintained long-term (>day 30 after negativization) for P1, P3, and P5. Figure 3 summarizes virological characteristics, alpha and beta diversity, and bacteria relative abundance for each participant. Specifically, P1 presented with multiple symptoms suggestive of COVID-19 and showed swab negativization at day 14 after the onset of symptoms. Symptom duration was 6 days and the stools were always negative for SARS-CoV-2.

According to Jaccard and weighted UniFrac, the structure of the gut microbiome underwent the most rapid change on day 1, when it reached the semi-static state lasting through day 15 after NP swab negativization; samples on the 30th day after negativization, however, suggested another state, different from both pre-COVID-19 and acute phase samples, suggesting the gut microbiome was undergoing further structural changes (Figure 3). The changes in the relative abundance of bacteria were detected during the active phase of the disease, with a decrease in beneficial symbionts such as *Faecalibacterium*, followed by an increase after swab negativization. Relative abundance of *Alistipes* and Parabacteroides, on the other hand, increased after COVID-19, with a marked increase in *Alistipes* from day 14 after swab negativization. *Aghatobacter*, *Prevotella*, and *Subdoligranulum* relative abundance was very low or undetectable compared with the pre-COVID-19 values. Alpha diversity was at its lowest during acute the phase of infection. However, it returned to the pre-COVID-19 levels 30 days after negativization.

P2 presented with multiple COVID-19 symptoms that lasted for 14 days; swab negativization occurred at day 22 and stools were positive until day 14 after symptom onset. Alpha diversity was at its lowest after swab negativization. The greatest changes in bacteria relative abundance were detected during the active phase of the disease, when greatest alpha diversity was also noted. Relative abundance of *Alistipes* showed a steep rise at day 14, while *Faecalibacterium*, *Bifidobacterium,* and *Streptococcus* decreased at day 7 after symptom onset. *Faecalibacterium* abundance returned to the pre-COVID-19 state, while *Bifidobacterium* and *Streptococcus* remained at lower levels compared to baseline. *Roseburia* and *Parabacteroides* increased over time compared to pre-COVID-19 levels. Overall, bacteria diversity and abundance were severely impacted by active disease but reverted, returning similar to pre-COVID-19 ones, as shown by weighted UniFrac and Bray–Curtis PCoA plots. P3 was the only participant with diabetes. He presented with anosmia, dysgeusia, and fatigue for 5 days. Swab negativization occurred at day 14 after the onset of symptoms. Stools were positive for SARS-CoV-2 until day 1 after swab negativization. Bacterial diversity progressively decreased during active COVID-19 until day 14 after swab negativization (as shown by Shannon’s) and then slightly increased from month 2 after negativization. UniFrac distance also showed a semi-constant state of the gut microbiome composition in the later stages of the acute disease until negativization. At day 30 after negativization, the composition was similar to the acute-phase one. A decrease in *Lachnospira*, *Faecalibacterium,* and *Roseburia* was recorded starting from day 14 from symptom onset. Similar to P1 and P2, an increase in *Alistipes* relative abundance was noted starting from day 14 after swab negativization. The gut microbiome composition was deeply affected by COVID-19. However, the observed changes in the gut microbiome were slower than in the other participants and did not appear to revert. P4 presented with transient fever and headache lasting only 2 days. A progressive improvement in alpha diversity and abundance of beneficial bacteria over time was observed. An increase in beneficial symbionts such as *Faecalibacterium* and *Lachnospira* and a decrease in *Bacteroides* and *Streptococcus* from day 1 to day 30 after swab negativization was noted. P5 presented with multiple symptoms lasting for at least 20 days, with full recovery occurring 4 weeks after symptom onset and swab negativization at day 21. Stools were positive for SARS-CoV-2 until day 1 after swab negativization. Alpha diversity was at its lowest at day 7 of active disease and progressively increased thereafter. The gut microbiome composition reached a semi-stable state between 1 day and 4 months after negativization. Relative abundance of *Bacteroides* and *Streptococcus* were high during active disease when *Faecalibacterium* abundance was low. *Alistipes* relative abundance increased following active disease. *Bifidobacterium* relative abundance increased and persisted after active disease, while *Faecalibacterium* increased only transiently. P6 presented with fever, anosmia, dysgeusia, and fatigue for 15 days, reporting a complete symptom resolution 40 days following symptom onset. Treatment with hydroxychloroquine was administered for 10 days. Swab negativization occurred at day 23 from symptom onset and stools were positive until day 2 after swab negativization. Alpha diversity reached its lowest 15 days after swab negativization and reverted only 4 months after disease. After increasing during active disease (e.g., day 14 vs. day 7), *Bifidobacterium*, *Roseburia*, and *Faecalibacterium* decreased. Parabacteroides and *Subdoligranulum* increased after swab negativization. *Alistipes* relative abundance decreased around day 14 from symptom onset and then increased steadily following active disease. Similar to P5, Bray–Curtis and weighted UniFrac PCoA plots showed gut microbiota changes mainly during COVID-19 compared to other timepoints.

**Figure 4:**
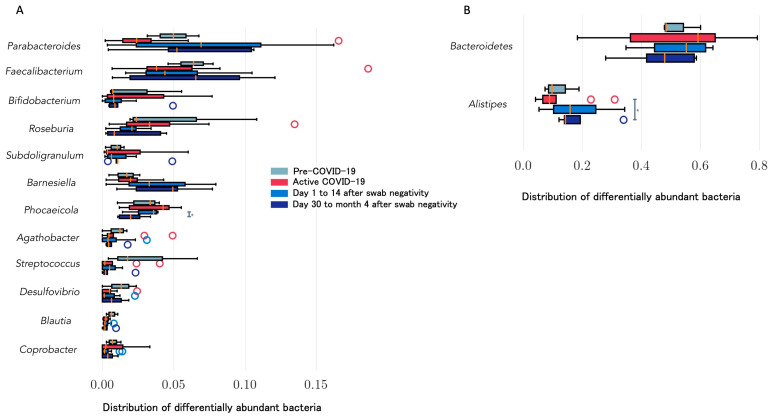
Comparison of bacterial relative abundances before and after SARS-CoV-2 infection according to low (**A**) and high (**B**) overall abundance. Relative abundance of pathogens with at least 3 values available for each time point are reported. Time points included pre-COVID assessment, active disease (e.g., day 7 and day 14 after symptom onset), post negativization (e.g., day 7 and day 14 following nasopharyngeal swab negativity for SARS-CoV-2), and follow-up (e.g., day 30, month 2, and month 4 following nasopharyngeal swab negativity). Asterisks indicate significant differences (*p* < 0.05).

A summary of bacterial abundance according to the timing of the disease (e.g., before COVID-19, during active COVID-19, from day 1 to 14 after swab negativization, and after day 14 from negativization) is reported in Figure 4. Bacteroides remained relatively stable among all subjects. *Alistipes* showed a significant increase from day 14 after swab negativization in all participants, while *Faecalibacterium* showed an increasing trend mainly during the late phases of active disease or after negativization. *Barnesiella* relative abundance increased, while *Roseburia* decreased starting from day 14 after swab negativization. Both *Agathobacter* and *Streptococcus* showed decreasing trends over time, while *Subdoligranulum* and *Coprobacter* increased during active disease, although differences between relative abundance over time were not significant.

Overall, we observed that high inflammatory levels largely corresponded to the low Shannon’s diversity index noted during active COVID-19 and up to 14 days after swab negativization compared with longer follow-up. Pre-COVID-19 analysis showed low levels of inflammation, and gut microbiota diversity was lower during active COVID-19 and up to 14 days after swab negativization. A minimum of 30 days following swab negativization was required to restore pre-COVID diversity levels, while inflammation levels showed partial recovery from day 30. *Faecalibacterium* relative abundance transiently decreased during active COVID-19, while *Alistipes* significantly increased from day 14 following swab negativization. This finding may be of interest as *Alistipes* has been previously associated with inflammation, immune dysregulation, and other clinical features including mental health and depression [29]. The study main limitation was represented by the low number of subjects enrolled; however, this was counterbalanced by the number of samples collected for each participant and by the availability of pre-COVID-19 samples. Moreover, our data belonged to a homogeneous cohort and were not biased by confounding factors such as hospitalization or antibiotic treatment; therefore, the changes observed in the gut microbiome composition reflected the sole effects of COVID-19.

## 5. Microbiome Changes and PCS

Although several studies have highlighted multiple alterations in the gut microbiota during the acute phase, these changes appeared often transient and might have been influenced by confounders, including medications and immune response to the virus. Studies are emerging to better understand gut microbiome alterations following COVID-19. PCS might present a complex array of symptoms that persist for several months after the initial viral infection has resolved. These symptoms can include fatigue, shortness of breath, chest pain, joint pain, cognitive impairment, and psychiatric complications such as anxiety and depression [30,31]. Studies investigating the relationship between PCS and gut microbiome changes are summarized in Table 2.

While previous studies of the gut microbiome in COVID-19 only hypothesized a connection between persistent microbiota dysbiosis after disease resolution and PCS, the first concrete evidence was provided by two Chinese studies published in 2021 [6,32]. A prospective study by Chen et al. (2022) monitored alterations in the gut microbiota of 30 patients with COVID-19 compared to 30 uninfected controls at three timepoints: acute phase (from illness onset to viral clearance), convalescence (from viral clearance to 2 weeks after hospital discharge), and postconvalescence (6 months after hospital discharge). The study found that gut microbiota richness, measured by Chao 1 index, was significantly lower in the acute phase of COVID-19 compared with uninfected controls. Although the authors did not directly compare patients with or without PCS, they found that reduced post-convalescence richness was associated with reduced pulmonary function of forced vital capacity, forced expiratory volume in the first second of expiration, inspiratory vital capacity, and total lung capacity. Although the sample size was relatively small and only pulmonary function was analyzed without considering other symptoms, these results suggested that persistent gut dysbiosis might be linked to PCS. The study also found that gut microbiota richness increased during convalescence but did not fully reach pre-infection levels up to 6 months after hospital discharge [32].

A direct comparison between recovered patients with and without PCS was performed by Liu et al. (2022). In this prospective cohort study, 68 controls and 68 hospitalized COVID-19 patients, 50 of which (74%) developed PCS, were compared. Stool samples were collected at admission and at months 1 and 6 after discharge. Bacteria diversity (Shannon index, Chao1 index) was lower in PCS vs. non-PCS patients and vs. controls at month 6 following acute disease. Interestingly, the same parameters were altered at admission in patients who developed PCS vs. controls. At 6 months, *Collinsella aerofaciens*, *F. prausnitzii,* and *B. obeum* were diminished, while *Ruminococcus gnavus* and *Bacteroides vulgatus* were enriched. The authors also investigated the association between PCS symptoms and microbiome signatures. Respiratory symptoms showed a positive correlation with opportunistic pathogens, neuropsychiatric symptoms, fatigue with nosocomial gut pathogens and persistent hair loss with depletion of BPB. An inverse association was shown between walking performance (e.g., measured using the 6 min walking test) and pathogenic bacteria, while a positive correlation was seen with bacteria producing SCFA and butyrate [6]. This study is particularly important as it demonstrated persistent lower bacterial diversity at 6 months after acute COVID-19 infection and due to the analysis of the association between microbiome composition and development of PCS-specific symptoms. The main limitation that is common to all these studies due to the difficulty in collecting longitudinal stool samples is the limited sample size.

Additional data were provided by Zhou et al. (2023). The authors analyzed stool samples collected from 15 patients 3 months after hospital discharge following COVID-19 and 14 healthy controls [33]. Persisting symptoms were reported by 80% of patients and included cough, fatigue, chest tightness after activity, and myalgia. The results confirmed significant alterations in fecal microbiota compared to healthy controls. Furthermore, *F. prausnitzii* was negatively correlated with chest tightness after activity (r = −0.591, *p* = 0.02), *I. butyriciproducens* negatively correlated with cough (r = −0.635, *p* = 0.011), and unclassified *Escherichia* positively correlated with fatigue (r = 0.567, *p* = 0.028), chest tightness after activity (r = 0.687, *p* = 0.005), and myalgia (r = 0.523, *p* = 0.045), while *Intestinibacter bartlettii* was positively correlated with anorexia (r = 0.629, *p* = 0.012) and fatigue (r = 0.545, *p* = 0.036). Despite the low sample size, which did not allow a direct comparison between patients with or without PCS, this study was the first to analyze the relationship between different microbial species and specific PCS symptoms, suggesting, for example, the association between the reduction in BPB (e.g., *F. prausnitzii*, *I. butyriciproducens*) and the persistence of respiratory symptoms such as cough and chest tightness after activity. Similarly, Vestad et al. (2022) investigated the alteration in gut microbiota composition as well as the levels of the gut barrier dysfunction marker lipopolysaccharide-binding protein (LBP) in patients with persistent pulmonary dysfunction at month 3 after COVID-19 compared with patients without persistent respiratory dysfunction [34]. A total of 83 patients were included, of which 25 (30%) showed persistent respiratory dysfunction defined as diffusing capacity of the lungs for carbon monoxide (DLCO) below the lower limit of normal. These patients displayed altered global gut microbiota composition by beta-diversity measures (e.g., Bray–Curtis) and reduced bacterial diversity by alpha-diversity measures (e.g., Faith’s phylogenetic diversity and observed amplicon sequence variants). Respiratory dysfunction was associated with increased relative abundance of five taxa and reduced relative abundance of twenty taxa, including *Erysipelotrichaceae* and several members of the *Lachnospiraceae* family and *Ruminococcaceae* family (*Subdoligranulum*, *Ruminococcus*), which are potential producers of butyrate. The authors also reported reduced *Erysipelotrichaceae* and increased *Veillonella* and *Flavonifractor*; furthermore, elevated LBP levels were significantly associated with several clinical markers of systemic inflammation and with impaired pulmonary function by inverse correlation with DLCO. This study is in line with Liu et al. (2022), showing altered bacterial diversity in patients with respiratory dysfunction 3 months after COVID-19 infection. A reduced abundance of BPB was shown, leading to the assumption of gut barrier alterations and a leakage of proteins like LPS that could influence the host immune system response to COVID-19, causing, for example, persistent low-grade inflammation. Even though this study adds interesting insights about the proportion of patients affected by persistent respiratory dysfunction, the authors did not specifically analyze patients with other PCS symptoms. Other limitations included the cross-sectional nature of the data (specifically, microbiota analysis and respiratory function tests were only performed at month 3) and the small sample size. Moreover, the results could have been affected by confounders such as the potential interaction with antibiotic use during hospitalization. Nonetheless, this is one of the first studies to potentially link pulmonary function to gut microbiota alterations in COVID-19.

Another group analyzed PCS and gut dysbiosis 12 months after SARS-CoV-2 clearance, with an average follow-up of 14 months [7]. A total of 155 patients were included and matched with 155 controls by age, gender, and body mass index. The prevalence of PCS was 79%, with the three most common symptoms being fatigue (51%), memory loss (45%), and difficulty in sleeping (36%). The study showed that, even beyond 12 months of follow-up, bacteria diversity and richness could be significantly lower compared with controls. Specifically, almost all symptoms among patients with PCS were associated with depletion of beneficial bacteria such as *Gemmiger formicilis* and *B. adolescentis*; moreover, most symptoms were associated with the enrichment of potentially pathogenic bacteria including *R. gnavus*, *Clostridium bolteae*, *Flavonifractor plautii,* and *E. ramosum*. The study confirmed long-term gut dysbiosis persistence following COVID-19. Moreover, specific alterations associated with PCS were identified and can be further studied to develop treatments or dietary interventions targeting the modulation of microbiota over time [7]. The same research group performed an investigation of the fungal and viral components of the microbiome, studying the interaction among the different microbial kingdoms (interactome) and the metabolome [35]. This cross-sectional and cohort study was conducted on 133 hospitalized patients with COVID-19. A total of 296 fecal samples (collected at admission, 3 months, and 6 months after discharge) were studied, including metabolomics on 79 fecal samples. Patients were divided in two clusters, based on their microbiome profile at admission: Cluster 1 included 63 patients (47%), and Cluster 2 70 patients (53%). Microbiome profiles were associated with clinical differences between the two groups: patients belonging to Cluster 1 were symptomatic at admission, had greater disease severity, and were more likely to develop PCS compared with Cluster 2 (84% vs. 44%, respectively). There was a significant difference in gut microbiome composition between the two groups: Cluster 1 had a lower diversity (both at admission at up to 6 months of follow-up) and an increase in opportunistic or pathogenic bacterial species, including *Erysipelatoclostridium ramosum*, *Clostridium bolteae,* and *Clostridium innocuum*. In Cluster 2, there was a higher total number of bacteria and a lower number of viruses. The interaction pattern among bacteria, viruses, and fungi was different between the two clusters. When the functional profiling of microbiome signatures was explored, the authors found that the urea cycle was enriched in Cluster 1 due to the presence of *Klebsiella* species [35]. Although the study does not specifically focus on PCS and microbiome, it is still valuable as it highlights how, for a more complete understanding of its pathogenesis, a more comprehensive microbiome analysis should be considered, including other kingdoms (fungi and viruses and potentially also archaea and protists), interactomes, and metabolomes [35]. In a smaller study, Carneiro et al. (2023) compared the gut microbiome of 19 patients with PCS with 15 non-PCS and did not find a significant difference in the gut microbiome composition [36]. Nonetheless, PCS patients had a lower ratio of an amplicon sequence variant (ASV) that is highly related to *Faecalibacterium prausnitzii* vs. other ASV related instead to *Bacteroides* species (e.g., *B. dorei*, *B. massiliensis,* and *B. thetaiotaomicron*) compared with controls. This finding provided an area under the curve (AUC) of 0.863 for differentiating patients belonging to the PCS group compared with the control group and was consistent with previous studies [6]. The main limitation of this study was the small sample size. In addition, the analysis was conducted on a single fecal specimen per patient and the timing of the sampling was not reported by the authors [36]. In a larger study, a Chinese group from Wuhan Union Hospital compared patients with PCS (*n* = 55) with patients recovered from COVID-19 who did not develop PCS (*n* = 75) and with a healthy control group (*n* = 32) [37]. Overall, 45% of patients who recovered from COVID-19 reported PCS-19 symptoms 12 months after discharge, most commonly cardiopulmonary symptoms, fatigue, myalgia, and gastrointestinal symptoms. The authors found significant differences in the gut microbial communities among the three groups; in particular, PCS patients showed significantly reduced diversity (Sobs and Shannon index, *p* = 0.002 and *p* = 0.003, respectively) compared with healthy controls. Furthermore, in the PCS group, a lower relative abundance of SCFA-producing symbionts (e.g., *Eubacterium hallii* group, *Subdoligranulum*, *Ruminococcus*, *Dorea*, *Coprococcus*, and *Eubacterium ventriosum* group) was displayed vs. controls. Conversely, *Veillonella* genus, belonging to the *Veillonellaceae* family, increased. Together with Su et al. (2023), this study provided the longest follow-up times and confirmed the presence of microbiome disruption even 12 months after acute COVID-19 infection. Moreover, a reduced presence of SCFA-producing bacteria in the PCS group was confirmed, as previously reported [6,7,32,33,34,35,36,37].

**Figure 5:**
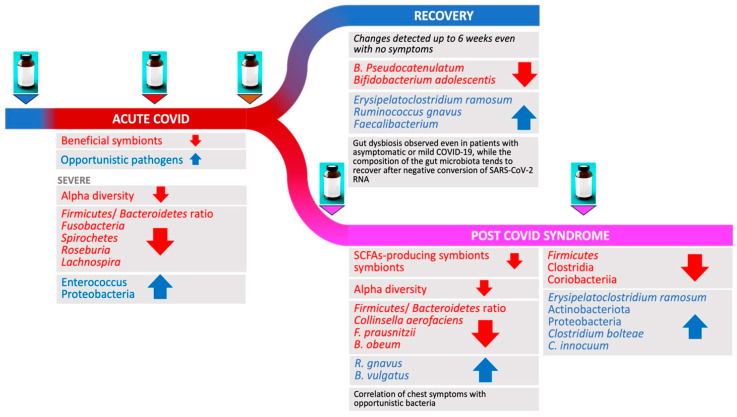
Most common gut microbiome changes (red arrow: increase; blue arrows: decrease) re-ported during SARS-CoV-2 infections, from acute disease to recovery or post-acute COVID syn-drome. The treatment icons represent the potential timing for intervention opportunities (e.g., before disease onset, as prevention; during acute disease; before and during post-acute syndrome).

A summary of the main gut microbiome changes reported during acute COVID-19 and PCS is reported in Figure 5.

## 6. Microbiome Manipulation and Treatment Potential

Interventions aimed at restoring gut microbiota homeostasis may have potential therapeutic benefits for COVID-19 patients. Probiotics (defined as live microorganisms which, when administered in adequate amounts, confer a health benefit on the host) [38], prebiotics (represented by a group of nutrients that are degraded by gut microbiota, producing SCFA) [39], and fecal microbiota transplantation (FMT) [40] have all been proposed as potential interventions to restore gut microbiota homeostasis in COVID-19 patients.

Administration of oral probiotics and prebiotics seems effective in exerting favorable effects on gut microbial diversity and composition which eventually can lead to better clinical outcomes in COVID-19 patients [3]. Clinical trials using dietary modification, probiotics, prebiotics, and FMT are planned or under way to determine their effectiveness in the treatment of acute COVID-19 and in enhancing the effectiveness of the SARS-CoV-2 vaccine. The rationale behind the manipulation of the gut microbiome to improve SARS-CoV-2 infection is complex and several authors have commented on the possibility that treating dysbiosis might alleviate COVID-19 complications, such as a leaky gut favoring gut mucosal protection or regeneration, or other metabolic-dysfunctions such as obesity which can cause severe and long-lasting COVID-19 [41].

It has also been shown that drugs that disrupt the gut microbiome, such as antibiotics, should be avoided during SARS-CoV-2 infection and may promote opportunistic infections while reducing symbionts [4]. Marked gut dysbiosis has been reported in patients with COVID-19 who have received antibiotics during hospitalization [42]. Furthermore, a longitudinal study including 200 patients showed that a reduced intake of antibiotics in the year prior to COVID-19 was associated with milder disease severity and more rapid clearance of SARS-CoV-2 [43].

Some studies advocated the potential of probiotics to interact with angiotensin-converting enzyme II, the host entry receptor of SARS-CoV-2) [44]. A pediatric RCT enrolling patients with ulcerative colitis showed that probiotics may alter the expression of IL-10 and reduce the expression of inflammatory cytokines [45]. Baud and colleagues in 2020 reported a list of potentially useful probiotics that might be able to reduce the burden of COVID-19, including *Bifidobacterium bifidum*, *Lactobacillus plantarum*, *Pediococcus pentosaceus*, *Leuconostoc mesenteroides*, *Bifidobacterium longum*, *Lactobacillus rhamnosus*, *Lactobacillus gasseri*, *Bifidobacterium breve*, and *Lactobacillus casei* [46], considering that *Bifidobacterium* and *Lactobacillus* appeared to be reduced in COVID-19 patients. In non-COVID contexts, prebiotics have shown an ability to reduce the levels of the proinflammatory IL-6, initially considered as the leading cause of the cytokine storm in patients with SARS-CoV-2 infections [46]. Din et al. (2021) conclude that the concurrent use of prebiotics and probiotics in COVID-19 infection could lead to several benefits, starting with decreasing the inflammatory response of viral pathogenesis and respiratory symptoms by strengthening the host immune system and improving the gut barrier function [47].

Furthermore, complex interactions exist between the different microbial components of both gut and lung microbiotas, reported as the gut–lung axis (GLA), following the concept that microbiotas of different body sites, such as the lungs, are essential for host homeostasis. The lungs and the intestine are linked through the mesenteric lymphatic system, which represents an essential pathway through which bacteria or metabolites, such as SCFAs, may translocate, overcoming the intestinal barrier and modulating the lung immune response [48]. The GLA can affect immune responses in respiratory diseases; for example, facilitating the building of immune memory through lung microbiota local antigens, or, conversely, lung microbiota can be morbidly affected by inflammation or infection [49]. The GLA may represent a potential target for therapeutic interventions aimed at modulating immune responses to face infections, including COVID-19.

Nevertheless, large-scale studies are lacking and there are no corresponding treatment guidelines yet. Therefore, while the therapeutic prospects for manipulating and restoring the gut microbiota in COVID-19 are promising, more research and larger trials are needed to confirm the potential efficacy.

Among RCT, the IMPACT study was a single-center, randomized, double-blind, placebo-controlled trial exploring the effects of gut microbiome modulation in reducing adverse health outcomes among 453 elderly and diabetic patients during the COVID-19 pandemic. The study used a novel microbiome formula, SIM01, on elderly individuals (≥65 years) and patients with diabetes mellitus [50]. The primary outcome was a composite of adverse health outcomes. Stool samples were collected at baseline and 3 months after the second vaccine dose for metagenomic sequencing and profiling. At baseline, there were no significant differences in the relative abundances of three probiotic *Bifidobacterium* species between the SIM01 and placebo groups. However, after the intervention, the SIM01 group exhibited a substantial increase in the relative abundances of these probiotic species. From a clinical point of view, subjects who received SIM01 had improved sleep quality (41% vs. 19%, *p* < 0.001) and better mood (21% vs. 11%, *p* = 0.043) compared to controls. The SIM01 group also displayed a slight but significant increase in the Shannon diversity index, indicating greater species diversity within the gut microbiome at 3 months compared to baseline. The trial contributed to the evidence that the probiotic formula had a positive impact on the gut microbiome composition, enriching probiotic species, increasing diversity, promoting beneficial microbes, and reducing detrimental pathogens [50].

Prior to this, Zhang et al. (2022) used SIM01 (oral encapsulated formulation of 3 lyophilized *Bifidobacteria*) in an open-label pilot study to evaluate the efficacy of SIM01 as an adjuvant therapy to impact immunologic response and the gut microbiota in patients hospitalized for COVID-19 [51]. Patients (*n* = 25) received two doses of SIM01 (100 billion CFU) per day, while controls were hospitalized but did not receive SIM01. At week 5, the levels of proinflammatory cytokines such as IL6, TNF-α, and IL-1RA decreased significantly in the SIM01 group vs. controls. The SIM01 group showed a significant increase in the abundance of the phyla Actinobacteria and Firmicutes, while there was a significant reduction in opportunistic pathogens such as *Escherichia coli* in the Proteobacteria phylum and *Bacteroides* spp. in the Bacteroidetes phylum at week 5.

Wischmeyer et al. (2022) examined the use of *Lactobacillus rhamnosus* GG as post-exposure prophylaxis for COVID-19 in 182 participants who had household exposure within 7 days. Probiotic use was associated with prolonged time to COVID-19 infection, reduced incidence of illness symptoms, and gut microbiome changes. However, the study was limited by a smaller-than-expected sample size, which limited its statistical power. Secondly, the study did not include vaccinated individuals [52].

Another study correlated *Bacillus subtilis* peptidoglycans with a reduced infectivity of SARS-CoV-2 [53]. Nevertheless, the impact of these findings has yet to be determined, especially considering the advent of new viral variants.

A randomized, quadruple-blinded, placebo-controlled trial was conducted to assess the therapeutic potential of a probiotic formula that included the *Lactiplantibacillus* plantarum strains KABP022, KABP023, and KABP033 and the *Pediococcus acidilactici* strain KABP021 in 300 COVID-19 patients [54]. This probiotic formula was well-tolerated and reduced nasopharyngeal viral load and lung infiltrates, as well as the duration of clinical symptoms (e.g., diarrhea, dyspnea, headache, and myalgia) compared with placebo. Moreover, the probiotic group had a higher serum level of virus-specific IgG and IgM than the placebo group. Probiotic treatment had no significant effect on gut microbiota composition. It was proposed that this probiotic formula might act on the GLA via interaction with the host immune system.

A retrospective, observational cohort study encompassing 200 patients with severe COVID-19 assessed the efficacy of *Bifidobacterium lactis* DSM 32246, *B. lactis* DSM 32247, *Lactobacillus acidophilus* DSM 32241, *Lactobacillus brevis* DSM 27961, *Lactobacillus helveticus* DSM 32242, *Lactobacillus paracasei* DSM 32243, *Lactobacillus plantarum* DSM 32244, and *Streptococcus thermophilus* DSM 32245 [55]. The study showed that the association of best available therapy (BAT) and oral bacteriotherapy could prevent disease progression in COVID-19 patients. Moreover, the mortality was lower in patients treated with BAT plus oral bacteriotherapy than those who only received BAT. A total of 375 COVID-19 patients were enrolled in a retrospective clinical trial to investigate the therapeutic efficacy of a probiotic combination that included *Bifidobacterium*, *Enterococcus, and Lactobacillus* [56]. Of these, 179 patients were treated with standard care plus probiotics. The administration of probiotics was correlated with improved clinical outcomes in COVID-19 patients, as evidenced by reductions in the duration of fever, viral shedding, and hospital stay.

Retrospective studies, usually with a limited sample size and few RCT, reported that administration of oral probiotics and prebiotics may induce positive effects in patients with COVID-19. However, the duration of these benefits is still unknown. Overall, more than 30 clinical studies are currently registered on ClinicalTrials.gov focusing on the use of probiotic species for COVID-19 treatment or prevention. Moreover, alternative strategies are under investigation and may also be of interest. An in vitro study using bacterial extracts obtained from human samples has identified three novel anti-SARSCoV-2 metabolites, namely N6-(Δ2-isopentenyl) adenosine, tryptamine, and 2,5-bis (3-indolylmethyl) pyrazine, which are structurally or functionally comparable to synthetic COVID-19 drugs such as remdesivir, fluvoxamine, and favipiravir [57]. FMT can also contribute to restore microbial dysbiosis via the transfer of a healthy microbiome to an individual with a disease and is used in the context of infectious diseases such as *Clostridioides difficile* infection recurrence [58]. Two reports have shown the use of FMT in COVID-19 [46,47,48,49]. Improved gastrointestinal symptoms were reported in five of eleven individuals with COVID-19 [59], along with increased abundance of *Bifidobacterium* and *Faecalibacterium*. In two patients with COVID-19 and concurrent recurrent *C. difficile* infection, FMT seemed safe and COVID-19-related respiratory symptoms rapidly resolved within 1 month [60].

In conclusion, probiotics, prebiotics, FMT and other strategies aiming at restoring the ecological homeostasis by regulating the gut microbiota are promising and represent an adjuvant approach to ameliorate or suppress COVID-19 severity. These interventions have been considered before disease occurrence (e.g., as prevention), during acute disease, and during PCS (Figure 5). Nevertheless, not only the efficacy but also the safety of probiotics in COVID-19 patients should always be considered and further RCT performed to ensure that their use does not induce new gastrointestinal symptoms or secondary infections.

## 7. Discussion and Conclusions

Our review highlights several relevant changes in gut microbiome composition that can occur during acute and post-acute phases of COVID-19. Acute changes during initial infection appear established after over 3 years from the beginning of the pandemic, following the general rule that opportunistic pathogens increase while beneficial symbionts decrease, thus favoring gut dysbiosis that is accompanied by reduced gut microbiome diversity. Some articles report specific signatures—or bacteria-based markers—that are typical of acute SARS-CoV-2 infection or even PCS. Although acute changes are similar in different studies and commonalities are evident even across different countries, there may be confounders that could undermine these conclusions or their generalizability. Firstly, data on COVID-19 severity are often not standardized and several severity scales are reported in different studies, sometimes based on organ failure, oxygen requirement, or ICU admission. Studies performed collecting stools before and after acute inflammation (e.g., the cytokine storm) or investigating parallel changes in immunities are limited. Secondly, treatments are often not listed or comparable among subjects either by duration or type of treatments, and several concomitant treatments can have a significant impact on the gut microbiome. Thirdly, samples are not always collected at similar timepoints from disease onset or from admission; therefore, the duration of hospitalization, occurrence of in-hospital bacterial colonization, or treatment duration can differentially affect the results in distinct groups or studies. Nevertheless, efforts have been made to match hospitalized patients from similar wards or to describe concomitant treatments, such as antibiotics that represent as well major disruptors of the gut homeostasis.

A paucity of studies, however, is available to longitudinally describe the gut microbiome changes in subjects with full recovery from the disease. The literature seems to highlight that major changes occur only during the first days of acute disease, with significant recovery soon after swab negativization and a steady progression reverting to a healthier gut. Nevertheless, some reports highlight changes that persist for weeks, even after mild or asymptomatic disease. In PCS, several studies include small numbers of subjects due to the difficulties in sample collection after 6 or 12 months of follow-up. Furthermore, changes in gut composition occurring after so many months can be affected by several factors such as new dietary habits, repeated SARS-CoV-2 infections or different viral variants, occurrence of novel infections or comorbidities, vaccination, and new treatments.

Despite these limitations, there are convincing data supporting long-lasting (>12 months) microbiome changes that often mirror acute COVID-19 dysbiosis with persistence over time. Nonetheless, long-term studies including more recent variants, for which different symptoms and limited numbers of hospitalizations have been recorded, are obviously lacking. Interestingly, some studies investigating PCS and the microbiome have analyzed the association between specific symptoms and gut microbiome signatures, highlighting that the occurrence of certain symptom clusters or PCS presentation could involve specific aspects of the gut–lung axis.

Studies on gut microbiome composition leading to “dysbiosis reports” or “healthy gut scores”, especially those based on thorough analysis such as WGS ones, are far away from being point-of-care tests due to the length of the analyses. Nevertheless, in the near future, gut microbiome signatures might contribute to predict disease severity or the evolvement of acute disease into PCT and could help in clarifying PCS pathogenesis or contribute to improving patient outcomes using personalized treatments that can affect specific targets or microbiome markers.

## Figures and Tables

**Table 1:** Characteristics of studies investigating microbiome changes in patients with acute SARA-CoV-2 infections and recovering from COVID-19.

Author, Country	COVID-19 Patients, Type of Study	Timing	Microbiome Changes	Measurement Method
Righi, Italy [4]	82 patients	Samples collected <2 weeks of hospitalization	16S rRNA gene sequencing. Increased opportunistic pathogens higher in severe COVID-19. *Enterococcus*: highest number of correlations with therapy (including antibiotics). *Dorea*, *Agathobacter*, *Roseburia*, and *Barnesiella* negatively correlated with antibiotic therapy	Alpha (Shannon’s index) and beta diversity metrics (Jaccard distance, weighted UniFrac distance)
Gaibani, Italy [20]	57 (32% ICU) and 100 controls	Cross sectional, case—control. Samples collected <week 1 of hospitalization	16S rRNA gene sequencing. Dysbiosis and over-representation of *Enterococcus*, especially in ICU; loss of alpha diversity	Alpha diversity metrics, number of observed ASV, and inverse Simpson index
Mazzarelli, Italy [21]	15 hospitalized patients (6 in ICU), 8 controls	Cross sectional, case—control. Swabs collected at hospitalization	16S rRNA gene sequencing. Patients in the ICU had lower microbial richness. Increase in *Proteobacteria* and decrease in *Fusobacteria* and *Spirochetes* in infected vs. controls	Alpha diversity and richness were assessed using Shannon indices and Chao1
Moreira-Rosário, Portugal [22]	Hospitalized and outpatients (WHO Clinical Progression Scale: 19 mild, 37 moderate, 59 severe)	Cross-sectional. Samples collected after enrolment	16S rRNA gene sequencing. Moderate and severe disease vs. mild had dysbiosis with lower Firmicutes/Bacteroidetes ratio, higher abundance of Proteobacteria, and lower abundance of *Roseburia* and *Lachnospira*	Shannon diversity index
Khan, India [23]	10 asymptomatic, 10 mild symptoms (no oxygen), 10 severe; 10 controls	Cross sectional, case–control. Samples collected at admission	16S rRNA gene sequencing. Decrease in diversity with increasing COVID-19 severity. Reduction in *Prevotella*, affecting the gut mucin glycoprotein maintenance, which interacts with the immune system	Alpha diversity and richness (InvSimpson and Fisher indices); Beta Diversity analyzed by LDA Effect Size
Reinold, Germany [24]	26 patients classified as severe, 12 as critical, 79 non-severe (WHO)	117 patients and 95 controls. Cross sectional. Timing of collection unknown	16S rRNA gene sequencing. Patients had lower richness with increased Proteobacteria and Bacteroidetes and decrease vs. control.	Shannon diversity index, observed features (ASVs), and Pielou’s evenness index
Newsome, United States [25]	94 patients (50 acute infection, 9 recovered, 34 controls ICU admitted)	Cross sectional, case–control. Samples collected <3 days of ICU admission	16S rRNA gene sequencing. Patients clustered in the recovered and non-infected groups. Recovered similar to non-infected. Similar Shannon index across groups	Shannon diversity index
Righi, Italy * (unpublished)	Healthcare workers (*n* = 6). Day 14 and >day 30 after negativization collection	6 to 20 weeks	16S rRNA gene sequencing. Bacteroides stable; *Alistipes* increase from day 14 after negativization. *Faecalibacterium* increased after negativization. *Barnesiella* increased. *Roseburia* decreased from day 14 after negativization	Alpha (Shannon’s index) and beta diversity metrics (Jaccard distance, weighted UniFrac distance)
Kim, Korea [26]	Two paired samples from 12 patients; negative conversion for SARS-CoV-2	10 days median interval	16S rRNA gene sequencing. Depletion of Bacteroidetes with tendency to rapidly reverse. Firmicutes/Bacteroidetes ratio higher in infected vs. recovered. Gut microbiota recovered after SARS-CoV-2 conversion	Faith’s phylogenetic diversity, Shannon’s index, and Pielou’s evenness

* Unpublished data. ASV = Amplicon sequence variants; ICU = Intensive Care Unit; WHO = World Health Organization; LDA = Linear Discriminant Analysis.

**Table 2:** Characteristics of studies investigating microbiome changes in patients following COVID-19.

Author, Country	Population, Type of Study	Timing	Microbiome Changes	Measurement Method
Liu, China [6]	68 patients with >1 symptom 4 weeks after SARS-CoV-2 clearance	6 months	16S rRNA gene sequencing. Bacteria diversity and richness lower vs. controls. Significantly lower *C. aerofaciens*, *F. prausnitzii, B. obeum* and higher *R. gnavus* and *B. vulgatus*.	Shannon index diversity and Chao1 richness index
Su, China [7]	155 patients (79% > 1 symptom 4 weeks after recovery from COVID-19)	14 months	Shotgun metagenomic sequencing Bacteria diversity (*p* = 0.004) and richness (*p* = 0.0003) lower than controls. Recovered patients had increased *E. ramosum* and *R. gnavus* and depletion of *B. adolescentis* and *B. pseudocatenulatum*.	Alpha diversity metrics (Shannon diversity, Chao1 richness)
Chen, China [32]	30 patients postconvalescence	6 months	16S rRNA gene sequencing. Richness lower in acute phase (median 217, IQR 164–266) vs. controls (median 432, IQR 332–468). Non-significant increase in Chao 1 index from acute to convalescence and postconvalescence phases. Low richness associated with reduced pulmonary function.	Chao 1 index
Zhou, China [33]	15 patients (12 with > 1 symptom)	3 months	16S rRNA gene sequencing. *F. prausnitzii* negatively correlated with chest tightness after activity and *I. butyriciproducens* with cough. Escherichia positively correlated with fatigue, chest tightness after activity, and myalgia. *I. bartlettii* positively correlated with anorexia and fatigue.	Spearman’s rank-based correlation test
Vestad, Norway [34]	83 patients (30% with respiratory impairment)	3 months	16S rRNA gene sequencing. Reduced alpha diversity. Increased abundance of 5 taxa and reduced of 20 taxa. Reduced Erysipelotrichaceae UCG-003 and increased *Veillonella* and *Flavonifractor*.	Alfa-diversity (Faith’s phylogenetic diversity and observed amplicon sequence variants). Beta diversity (Bray–Curtis)
Liu, China [35]	78 (Cluster, 42; Cluster 2, 36) > 1 symptoms at 4 weeks after infection	6 months	WGS analysis. Cluster 1 (84% long COVID) different vs. Cluster 2 (44% long COVID). Bacteria diversity Cluster 1 lower than Cluster 2. Cluster 1 had increased opportunistic species (*E. ramosum, C. bolteae, C. innocuum*).	Shannon diversity index, MaAslin analysis
Carneiro, US [36]	34 patients, 15 with continuing or recurring symptoms > 4 weeks after infection	N/A	16S rRNA gene sequencing. No significant differences between groups. Long COVID had lower ratio of ASV highly related to *F. prausnitzii* over genus Bacteroides (*B. dorei, B. massiliensis*, and *B. thetaiotaomicron*).	SELBAL analysis
Zhang, China [37]	55 patients with >1 symptom	12 months	16S rRNA gene sequencing. Decreased richness and diversity; Firmicutes less abundant, Actinobacteriota and Proteobacteria more abundant; decreased Clostridia and Coriobacteriia. SCFAs-producing symbionts significantly depleted. *Veillonella* enriched.	Sobs and Shannon index, PCoA of Bray–Curtis

ASV = amplicon sequence variant; PCoA = Principal Coordinate Analysis.

## Supplementary Materials

The following supporting information can be downloaded at: https://www.mdpi.com/article/10.3390/microorganisms12010131/s1, Table S1: List of bacteria with pro-inflammatory, not clear pro-inflammatory or anti-inflammatory, and anti-inflammatory profiles; Table S2: List of main categories of diseases included.
