# Supplementary material for: Gut Microbiome Disruption Following SARS-CoV-2: A Review
Source: Microorganisms. 2024 Jan 9;12(1):131. doi: 10.3390/microorganisms12010131 (PMC10820238; doi:10.3390/microorganisms12010131)
Supplement: Supplementary file 1 [file microorganisms-12-00131-s001.zip › microorganisms-2706579-supplementary.pdf]

Supplementary Materials

Table S1. List of bacteria with pro-inflammatory, not clear pro-inflammatory or anti-inflammatory, and anti-inflammatory profiles. The missing taxa are a result of the lack of sufficient evidence in the Pubmed search.

**Pro-Inflammatory**	**Not Clearly Pro-Inflammatory or Anti-Inflammatory**	**Anti-Inflammatory**
Parabacteroides<br>Alistipes<br>Bacteroides<br>Desulfovibrio<br>Streptococcus<br>Balutia<br>Dorea<br>Parabacteroides<br>Bacteroides<br>Pseudomonas<br>Ruminococcus	Agathobacter<br>Barnesiella<br>Coprobacter<br>Holdemanella<br>Paraprevotella<br>Phocaecola<br>Subdoligranulum<br>Turicibacter<br>Lachnoclostridium<br>Maihella<br>Haemophilus<br>Prevotella	Roseburia<br>Faecalibacterium<br>Bifidobacterium<br>Akkermansia<br>Lachnospira<br>Oscillospira

Table S2. List of main categories of diseases included.

**Category Used for the AI/PI Score**	**MESH Terms Reported in PubMed or Abstract of the Publication**
IBD or Inflammation in the gut	Inflammatory Bowel Diseases<br>Irritable Bowel Syndrome<br>Pain with Irritable Bowel Syndrome<br>Crohn Disease<br>Crohn Disease with ASCA<br>Crohn Disease with injury<br>Inflammation<br>Intestinal Diseases<br>Gastroenteritis<br>Gastrointestinal Diseases<br>Colitis<br>Colitis, Ulcerative<br>Diarrhea
Cardiovascular Diseases or hypertension	Atherosclerosis<br>Cardiovascular Diseases<br>Chronic heart failure

	Hypertension<br>Blood pressure
Autoimmune Diseases	Autoimmune Diseases,<br>Multiple Sclerosis,<br>Systemic sclerosis (SSc)<br>Amyotrophic lateral sclerosis
Metabolic Diseases	Metabolic Diseases<br>Metabolic Syndrome
Renal diseases	Renal disease<br>End stage renal disease<br>Diabetic nephropathy<br>Renal Insufficiency, Chronic
Liver Diseases	Liver Diseases<br>Liver Cirrhosis<br>Non-alcoholic Fatty Liver Disease<br>Cystic Fibrosis with Liver Cirrhosis
Diabetes or insulin resistance	Diabetes,<br>Insulin resistance<br>Diabetes Mellitus<br>Diabetes Mellitus, Type 1 and Type2<br>Gestational diabetes mellitus<br>Obesity with diabetes
Cancer, gastrointestinal tract	Colonic Neoplasms<br>Colonic Polyps<br>Colorectal Neoplasms<br>Colorectal cancer<br>Stomach Neoplasms<br>Neoplasm'
Depression or Bipolar or Schizophrenia or Stress	Depressive Disorder<br>Depressive Disorder, Major,<br>Bipolar Disorder<br>Schizophrenia<br>Anxiety<br>Depression<br>Stress, Physiological<br>Stress, Psychological<br>Mental Disorders<br>Anxiety
